# Citizen Science Studies in Nursing: A Systematic Review

**DOI:** 10.3390/nursrep14020072

**Published:** 2024-04-16

**Authors:** Carmen Torró-Pons, Carlos Saus-Ortega, María-Luisa Ballestar-Tarín

**Affiliations:** 1 Department of Nursing, Faculty of Nursing and Podiatry, University of Valencia, c/Jaume Roig s/n, 46010 Valencia, Spain; carto5@alumni.uv.es (C.T.-P.); m.luisa.ballestar@uv.es (M.-L.B.-T.); 2 Grupo de Investigación Arte y Ciencia en Cuidados, Escuela Universitaria de Enfermería La Fe, IISLaFe, Av. Fernando Abril Martorell, nº 106, 46026 Valencia, Spain; 3 Nursing Care and Education Research Group (GRIECE), GIUV2019-456, University of Valencia, c/Jaume Roig s/n, 46010 Valencia, Spain

**Keywords:** citizen science, nursing, health

## Abstract

Background: Citizen science is a research approach wherein citizens actively participate alongside professionals in some or all stages of the research process. The bidirectional benefits it generates, especially in the field of health, including empowerment, new hypotheses, and results, and addressing issues truly important to society, justify the necessity to establish a common framework and address barriers to ensure a fruitful evolution of this new approach within nursing research. The aim was to analyze nursing projects with a citizen science focus that have been conducted. Methods: PRISMA guidelines were employed to conduct a systematic review. Searches were conducted on PubMed, CINHAL, LILACS, IBECS, and Cochrane. Following the identification and screening process, 13 studies were included. The quality of the articles was assessed using the Joanna Brigg Institute (JBI) critical appraisal checklist and the quality of citizen science research using the Citizen Science Appraisal Tool. Results: Citizen science studies in nursing were notably recent (2017–2023). Five research areas were identified, with environmental health being the most predominant. Multiple tools, both technological and traditional, were utilized, with the “Photovoice” and “Our Voice” methodologies being prominent. Citizen participation was limited to data collection and analysis in 7 out of the 13 studies, with most studies working with small samples. Findings regarding the application of this practice were positive, but no study exceeded 26 points on the CSAT scale to be considered high quality in citizen science. Conclusions: Citizen science can be a promising approach within the field of nursing. There is a need to increase individual participation to fully realize the potential bidirectional benefits. It is imperative to establish a common theoretical framework and continue working on the development of this methodology within nursing.

## 1. Introduction

Citizen science (CS) has multiple definitions derived from its transdisciplinarity and integration in multiple sciences such as natural, physical, humanities, social sciences, and recently, health sciences [1,2]. The earliest definitions of this concept emerged in the mid-1990s simultaneously in the United Kingdom and the United States, spearheaded by Alan Irwin and Rick Bonney, respectively [3]. While more recently, these initial ideas could be aligned with a newer definition by Rowbotham, describing it as the general public’s participation in some or all stages of the scientific process in collaboration with researchers or professional scientists [4]. This type of research approach differs from other participatory approaches in that, due to individuals’ collaboration, it extends beyond the data collection process and involves the true immersion of citizens in scientific activities [3]. The early CS studies were related to bird migration or tide prediction [5]. It was not until 2008 that this approach experienced significant growth globally in recent years [5,6]. Technological advancements, easy access to information, and the increasing public awareness of the importance of science and research could be reasons for its development [6,7]. In addition, the CS approach allows to address complex problems from multiple perspectives [1,2]. 

This research approach offers benefits for both citizens and scientific professionals [8]. Citizens, on the one hand, experience empowerment through the opportunity to take on responsibilities and addressing relevant problems [9], while on the other hand, they develop scientific skills [8,10], making it an educational tool with formative and awareness-raising impact [11]. As far as scientists are concerned, CS projects drive new questions, hypotheses, and increased participation, facilitating greater research capacity in a more efficient manner [12]. Furthermore, within the realm of health, CS provides a fresh perspective on research, enabling a much more comprehensive approach that incorporates individuals’ experiences, sensations, and knowledge, thereby overcoming some of the limitations of more traditional studies such as participant dropout, abandonment, or the lack of personal and financial resources [13,14,15].

However, the main limitations of these studies focus on ethical aspects. Issues such as copyright, intellectual property, confidentiality, commitment, respect, and inclusion of individuals or groups must be addressed [9,16,17]. Additionally, there is uncertainty about the validity of the information obtained through CS tools, which is associated with the misconception of lack of scientific rigor [16]. Other limitations include project logistics, encompassing training, time, and resources [18,19], as well as the public’s perception of science as manipulable [20]. This approach shares limitations with traditional studies, which also require motivated participants, considering factors such as standard of living, time, or lack of knowledge [21,22]. 

Another challenge of CS is the lack of a unitary taxonomy [23]. Rowbotham et al. propose three levels of CS based on the level of citizen participation: (a) contributory, which involves citizens solely in the data collection process; (b) collaborative CS, where participation extends to the analysis and interpretation of data; and (c) co-created QC, where citizen participation is maximized by including them in the definition of the problem or carrying out the transmission and impact of the results [4]. However, there are broader classifications of CS [9,24]. In this classification, the three previous levels are supplemented with the following: (a) Contractual projects, where individuals are the ones who suggest research to scientists. These fall between contributory and collaborative CS. (b) Independent projects, where citizens conduct the entire project. The maximum level of participation is reached here [9,14,24]. King et al. also classified CS studies into three levels according to participation: (a) for individuals, conducted entirely by scientists, with passive collection of citizen data; (b) with individuals, when citizens and scientists work actively in some of the stages, generally data collection; and (c) by individuals, where there is joint work practically in its entirety [25]. Furthermore, according to Roy et al., studies can be classified according to the number of participants as follows: (a) “local”, carried out with community groups with a smaller number of participants, and (b) “massive”, involving a large number and open to all sectors of society [26]. 

The application of CS in the field of health is a relatively recent phenomenon compared to other fields, such as environmental sciences, and which has experienced remarkable growth in recent years [27,28]. The field of health ranges from health promotion (communication and participatory health literacy) [29] to biomedicine, occupational health, and environmental health [30,31,32]. This new research paradigm seeks to provide citizens with the knowledge and information necessary to protect and improve their health, playing a crucial role in disease prevention [29,33]. This research approach provides a more immersive perspective of citizens’ experiences and knowledge, enabling a more comprehensive understanding of health–disease processes [13,14,15]. All of this can motivate researchers to explore certain scientific questions in a more inclusive and effective way [13,34]. In this regard, in the 2016 report of the Spanish Observatory of Citizen Science, when referring to the Community Nursing Association and its participatory methods, the importance of an informed, responsible, and actively collaborating patient is highlighted. All these initiatives emerge with the purpose of facilitating effective interaction between active patients and healthcare professionals [6]. In the field of nursing, the magnitude and origin of this approach are still unknown. Hence, the following research question is posed: what are the nursing projects with a citizen science approach that have been carried out? Therefore, the aim of this study was to analyze the citizen science-focused projects developed within the nursing context.

## 2. Materials and Methods

### 2.1. Design

A peer-reviewed systematic review was conducted between September and October 2023 following the recommendations of the PRISMA 2020 guidelines [35] The entire review process was conducted by peers, and in case of discrepancies, a third investigator was consulted.

### 2.2. Search Strategy

Two researchers, independently (C.T.-P. and C.S.-O.), conducted structured searches in the following electronic health databases: PUBMED, CINHAL (Cumulative Index to Nursing and Allied Health Literature), LILACS (Literatura Latinoamericana y del Caribe en Ciencias de la Salud), IBECS (Índice Bibliográfico Español en Ciencias de la Salud), and Cochrane. 

The MeSH (Medical Subject Headings) terms identified were as follows: “Citizen Science”, “Nurse”, “Nursing”, using the Boolean operator AND. No limitations were placed on the time period to capture as many relevant articles as possible. In case of discrepancies in the search process, a third investigator (M.-L.B.-T.) was consulted.

### 2.3. Inclusion and Exclusion Criteria 

All articles that met our objectives were included, without limitation on publication date, and published in English or Spanish. To be included, articles had to explicitly state that they had used citizen science as a research methodology at any level of participation and be related and categorized as projects within the nursing field. Those that spoke of other collaborative approaches such as community participation or crowdsourcing were excluded. Additionally, review articles, comments, or conceptual presentations of the topic were also excluded.

### 2.4. Study Selection Process

**Figure 1:**
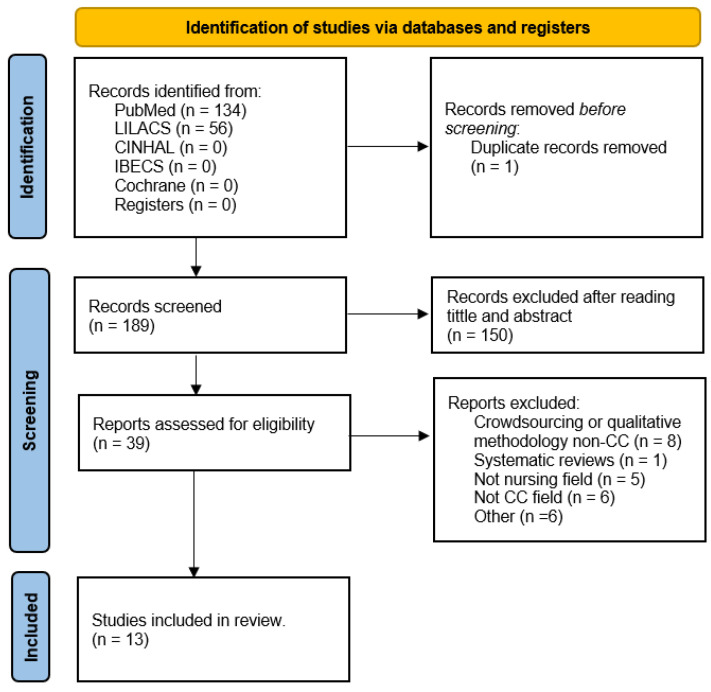
PRISMA flowchart with the search strategy of the systematic review [35].

Two investigators carried out the study selection process independently in various phases, consulting with a third investigator in case of any doubts. In the first phase, 190 articles were identified, and one was eliminated due to duplication. In the second phase, the titles and abstracts were read in full (N = 39); based on the title and abstract reading, another 150 studies were excluded, leaving 39 investigations that were read in full. Among these, 8 were excluded as they were another type of research approach, 10 were excluded as systematic reviews, 1 as non-nursing, and 6 were excluded as they were meetings, expert discussions, meta-evaluations, and other similar methodologies. Thus, a total of 13 articles were included in this review. The results obtained in the selection process are presented in Figure 1 following the PRISMA 2020 guidelines.

### 2.5. Quality Assessment of the Studies

The quality assessment of the articles included in our review was verified using the critical appraisal checklist through the Joanna Briggs Institute (JBI) qualitative review assessment tool [36]. This checklist allows the methodological quality of a study to be assessed and determines the extent to which bias has been addressed in the design, conduct, or analysis. Articles were scored based on the sections where they scored positively in the critical appraisal and the final score was calculated.

### 2.6. Analysis of the Quality of the Citizen Science Research Approach in the Studies

The Citizen Science Appraisal Tool (CSAT) is a tool developed by the University of Birmingham to assess the quality of citizen science and other participatory approaches [37]. This instrument takes into account, for quality analysis, aspects such as participation, objectives, results, future impact, dissemination, and data quality, among many others, which are key elements in determining high-quality citizen science.

It works through a scoring system and assesses it based on three systems of citizen engagement: contributory, collaborative, and co-created. It consists of a total of 16 items distributed within 4 sections (science and research; leadership and participation; delivery and data; and outcomes, evaluation, and open data). Each question is scored based on the evaluator’s ability to know the answer with the following: yes = 2 points; unclear = 1; and no = 0. The scores are then summed up and translated into a final score. Scores are considered low between 0 and 6 points, low-medium (7–12), medium (13–19), medium-high (20–26), and high (27–32). This score shows the quality of the study. The questionnaire was used to assess all included studies (*n* = 13).

## 3. Results

This review was conducted following the PRISMA statement, in accordance with the set of guidelines for conducting reviews and meta-analyses in a comprehensive and systematic manner it provides [35].

In the first search process for citizen science (CS) and nursing, a total of 198 articles were identified. Through the reading of titles and abstracts, 150 articles were excluded for not meeting the inclusion criteria, leaving 39 studies selected. The full text of the 39 articles was retrieved and critically appraised. During this process, 25 articles were eliminated, 10 for being another type of participatory research approach, 1 for being a systematic review, 4 that did not belong to the nursing field, 5 that did not belong to the field of CS, and 6 for being meetings, expert discussions, and other methodologies of a similar nature, leaving a total of 13 studies included (Figure 1). 

### 3.1. Methodological Quality Evaluation

The 13 studies included in this review presented a qualitative methodology, and the Joanna Briggs Institute (JBI) critical appraisal checklist was applied to assess their quality. The results of the assessment are shown in Table 1. No article was excluded from the review due to quality issues. Assessment criteria 1, 2, 5, and 10 of the JBI checklist were met by all articles. Criterion P7 is not present in 7 out of the 13 articles, and criterion P9 is missing in 6 of the articles included in the review. The remaining criteria are not met in 1 to 3 articles.

### 3.2. Study Characteristics

The included studies were published between the years 2017 and 2023. They have been conducted in the United States of America [38,41,43,44,47,48,49,50], Australia [39,45,46], France [40], and South Africa [42]. The qualitative methodology of data collection included different techniques, such as Photovoice [38,47], audio recordings [39,42,46], discussions [50], or questionnaires [44,49]. Table 2 presents the characteristics of the studies analyzed.

### 3.3. Nursing Study Areas with a Citizen Science Focus

Nursing research with a citizen science (CS) focus was grouped into five areas: environmental health [38,43,44,47], environmental improvements [39,42,45], food safety [41,46,50], chronic diseases [40], and COVID-19 [48,49]. However, within these categories, there are diverse lines of inquiry.

The environmental health area was the most predominant with 4 out of the 13 selected articles [38,43,44,47]. This group includes Hann, E. et al. and their project on radon exposure, where citizen scientists participated in various stages of the process such as sampling in homes [43]. Similarly, Brickle, M. et al. used citizen science to address issues arising from wood smoke pollution in communities [38]. Evans Agnew, R. A. et al. worked with a group of young people to examine exposure to volatile organic compounds in households [47].

In the food safety area, 3 out of the 13 studies were included [41,46,50]. D’Alonzo et al. used this approach to address the problem of obesity among a Mexican immigrant population [50], while Kim et al. and Tuckett et al. used this methodological approach to understand and improve the food security of two completely different populations, young people and older adults, respectively [41,46]. 

Within the environmental improvement area, we find the case of Rowbotham et al. who studied through citizen science the circumstances that women encounter in their work environment that facilitate or hinder the possibility of continuing breastfeeding [45]. Meanwhile, Odunitan-Wayas et al. and Tuckett et al. used citizens’ efforts to address various elements of the environment that hinder or promote physical activity in a low-income population and in a group of older adults, respectively [39,42].

The remaining articles were included in the areas of chronic diseases [40] and COVID-19 [48,49]. Regarding chronic diseases, it is not specified which ones are being studied, but citizen scientists, in this case patients, are used to generate ideas to improve medical care and meet their needs [40]. In the case of COVID-19, both cases focus on symptomatology, in one project through women [48] and in another working with an elderly population [49]. 

### 3.4. Data Collection Tools and Methodologies

The tools used for data collection in nursing studies with a CS approach were grouped into two categories: technological tools and traditional tools. 

Among the technological tools, mobile applications were the most commonly used instrument for data collection, with 6 out of the 13 selected studies utilizing them [39,41,42,46,48,49]. The two studies published by Tuckett et al. [39,46] and also the one published by Odunitan-Wayas et al. used a mobile application called the Stanford Neighborhood Discovery Tool [42], a computerized participatory tool designed at Stanford University originally intended to help residents identify neighborhood features that affect their active life. This tool allows citizen scientists to upload photographs and audio narratives that can later be analyzed to work on findings collaboratively [51]. On the other hand, other authors used the CovidWatcher application, through which only user-recorded data on the symptomatology of COVID-19 is stored [48,49]. Another technological tool used was online surveys through various online platforms [40,45]. Also, within this block, cameras were used to collect data and visual information that was then discussed in the debates [38,45,47]. Finally, in Rowbotham et al.’s study, social networks were used, specifically Facebook, a platform through which the women participants could upload photos and write comments that the researchers would later also include in the analysis [45]. 

As far as traditional tools are concerned, one of the most commonly used was face-to-face surveys, with 2 of the 13 studies utilizing it [41,43]. Kim et al.’s case stands out for using this tool due to the lack of internet access for the use of the mobile application initially proposed [41]. Data collection also included group discussions and debates [44,50]. Finally, data were also obtained through actual measurements, such as radon levels from the study by Hahn et al. [43], and air quality values measured by Brickle et al. and Evans-Agnew et al. [38,47].

In terms of methodologies, the Our Voice method was common in the areas of food security and environmental improvement, carried out in different populations [39,46]. It is a global research initiative, based on technology and committed to the community, aiming to empower residents from various locations to capture and enhance their environments, both physical and social, through various methods, modifying those characteristics that may affect various aspects of health [4]. The Photovoice method was also used, involving communities in dialogue and action for social change through photographs [38,47]. 

### 3.5. Participation and Inclusion of Citizen Scientists in the Research Process

Nursing research with a citizen science approach was grouped according to the classification proposed by Rowbotham et al., from lower to higher participation, into contributory, collaborative, and co-created.

In the contributory category, it was found that 4 out of the 13 articles limited citizen participation to the data collection process [44,45,48,49]. Data were collected through different tools, and researchers analyzed the data and presented the results. In the second level, there were 3 studies classified as collaborative [42,43,50]. In these investigations, citizens were able to participate, and their learning about data interpretation, prioritization, and analysis was facilitated.

Finally, we find the co-created projects, the ultimate expression of citizen science. However, it was observed that many of them were on the border between collaborative participation and a co-created project. Six of the 13 selected studies were part of this group [38,39,40,41,46,47]. None of the articles included citizens in defining the problem; therefore, none of them could be considered 100% co-created. Nevertheless, studies by Tuckett et al., Brickle et al., Kim et al., and Evans-Agnew et al. were included, as their participants were involved in the data collection, analysis, discussion, and dissemination process among their communities [38,39,41,46,47]. In addition, participants learned how to use their findings to interact with the rest of the public, as well as representatives or political figures capable of initiating change [38,39,41,46,47]. Only in the article by Tran et al. were participants invited to be authors of the manuscript and worked on the review process before publication [40].

### 3.6. Analysis through CSAT

Table 3 shows the evaluation of the quality of the citizen science approach applied in each research. No study exceeds 26 points to be classified as high-quality citizen science. All were between the medium-low (7–12 puntos) and medium-high levels (20–26). Tuckett et al. and Tran et al. achieved the best results with 26 points [39,40]. 

The scores obtained are not related to a higher quality of the study, but are intertwined with the degree of citizen participation and the quality of the CS applied. For this reason, we observe that studies with a contributory focus [44,48,49] correspond to the lowest scores ranging between 11 and 12 points, as citizen participation is limited to data collection. The highest scores, between 24 and 26 points [38,39,40,41,46,47], are related to studies considered as co-created, where citizen participation is practically in all stages of the research process, considering these studies of higher quality in terms of citizen science. 

There are some exceptions such as the case of Rowbotham [45] which despite being classified as a contributory project obtained a score of 21, this being due to the influence of questions on this scale that, in addition to participation, assess other determinants of a good CS project. This includes the definition of objectives and functions in dimension 1, consideration of limitations in dimension 3, or the clear presentation of results and their relationship with scientists.

## 4. Discussion

This review presents research with a citizen science (CS) approach in nursing. The works were grouped into five areas, with environmental health and food security projects being the most prominent. For data collection, these studies mainly used electronic technologies such as mobile apps. Contributory and collaborative CS approaches were the most predominant, although practically co-created studies were also found. The quality of these latter CS approaches was medium-high on the CSAT scale.

Regarding geographical distribution, most published studies were conducted in the United States, followed by other locations such as Australia and Canada. These findings are consistent with other reviews such as those by Marks et al. and Rosas et al. [13,14]. As for the temporality of the studies, most citizen science (CS) research in the health domain is relatively recent, showing growth in the last 10 years. It is also observed that although the integration of CS as a research model is relatively recent in the field of health, it has experienced exponential growth in recent years. The oldest study included in this review is from 2017 [38]. These results are in line with other reviews [14,16,52,53], and coincide with the establishment of institutions such as the Australian Citizen Science Association in 2014 or the European Citizen Science Association in 2015 [54]. 

Regarding the thematic areas of research, our search highlighted environmental health, as well as environmental improvement and food security. These results align with evidence describing environmental sciences as the research area where the most citizen science projects have been launched and with those that position it as the origin of this methodology [6,15,55]. As for environmental improvement, there are projects that propose similar objectives such as developing or implementing interventions within a community, also intertwined with the themes observed in this line with projects on physical activity or green and walkable spaces [14]. Food security was another strong focus in this project, as in the review published by Marks et al. [14]. 

Online tools have currently become researchers’ best allies in citizen science (CS) projects. This aligns with what Schaaf et al. have presented regarding the use of computer systems for research [56], mainly participation through mobile or web applications. This is the case of CovidWatcher [49] and other specific citizen science applications like the Standford Healthy Neighborhood Discovery Tool, used in two out of the thirteen selected projects [39,42]. Moreover, online tools, according to Scheibein, are a clear facilitator for the implementation of CS and other similar types of science such as open science or crowdsourcing in new projects [16,53]. These tools include the use of cameras and mobile applications, online surveys and questionnaires, or different websites that are accessible to users for free or for a fee [53,57]. It was evident from numerous studies, as well as those published by Brickle et al. in our review, that Photovoice was one of the most popular methods in this type of research, followed by the method encompassed within the “Our Voice” tool developed by Stanford University [38,39,53].

In this regard, mobile applications and technology in general serve as facilitators for the implementation of these projects. According to Brickle et al., the implementation of these methods and the use of new technologies are particularly appealing to young people, with benefits such as being able to express their opinions through the use of photographs [38]. Several included studies express the suitability of using technology to engage citizens in research [42,48,49]. These assertions are consistent with other authors who report benefits such as facilitating data collection on a large scale, enhancing citizen participation, and educating individuals in various fields of research [58,59,60]. However, as highlighted by Bayih et al., it is important to address economic and social disparities in some populations that hinder the implementation of these tools [58].

As far as the sample of individuals is concerned, most of the studies included in this review had samples of between 8 and 37 participants. Findings that align with the evidence found, such as the case of Marks et al., where in their publication on citizen science and chronic diseases, most studies had no more than 50 participants [13,14]. This is closely linked to the involvement and inclusion of citizen scientists, where small-scale studies are much more ambitious in the CS approach and give more prominence to the non-scientific population. Marks et al. also add, in accordance with other authors, that CS projects tend to be conducted within narrow geographical boundaries such as neighborhoods, districts, or cities [14,61]. In our study, the samples are notably larger when individuals only participate in the data collection process. This is also the case with the findings of a similar review on mental health published by Todowede et al. They report how in most cases participation was limited to this stage [52]. Derived from this, it is discussed in several articles that there is a directly proportional relationship, where greater participation is also associated with more benefits [52,62]. 

Most studies conclude that CS is a viable and valuable tool for health research, as well as a facilitator for the presentation of new problems and solutions [39,46]. This opinion is shared by other authors such as Gjoneska et al., who suggest that CS provides benefits in fields with emerging research, where the involvement of non-scientific individuals can increase visibility, facilitating the process of prioritizing and planning research [17]. 

One strength of using this approach is the ability to identify the health needs of the population and streamline the process for implementing improvements in care [40,48,49] This could be due to the possibility of working collaboratively with a large amount of data in a reasonable timeframe [27,57,63]. An example of this can be seen in the study by Gjoneska et al., which focused on improving chronic disease management, mental well-being, and increased adherence, participation, and prevention of numerous diseases [17]. On the other hand, one cannot forget the increased involvement of individuals in the management of their own and their community’s health, empowerment, and the ability to influence policy decisions [9,64]. Additionally, there is an increase in skills and a better understanding of scientific work, and the topics addressed in these projects [8,10]. Overall, CS promotes public acceptance of research and a noticeable increase in credibility, promoting public evaluation of research, which ensures basic standards for projects and ensures that they are valid and applicable within their context [9]. Among the benefits it brings, it is important to highlight the formation of social capital and citizen inclusion in local issues [65].

However, citizen science (CS) also faces numerous challenges shared by various authors. Some of the studies included in this article discuss the challenge posed by the recruitment process and the training of individuals, the necessary economic resources, and the obtaining of reliable results due to the possible influence of the scientific researcher or other citizens [41,43,44]. These limitations, along with others, are shared by other authors who refer to challenges related to data such as objectivity or representativeness, as well as limitations regarding citizen participation, such as opportunity asymmetry, lack of interest, or other barriers to involving groups [65,66]. One of the major challenges presented by this approach is all the ethical issues surrounding it. All ethical issues such as copyright, intellectual property of projects, or individuals’ confidentiality must be addressed, as well as engagement, respect, or inclusion of certain individuals or groups [9,16,17]. 

Although the studies generally show high quality, the existing limitations demonstrate that projects including a small sample size face the challenge of the impossibility of conducting inferential statistical analysis, limiting the generalization of results internationally [38,41,42,45].

### Limitations of this Review

Due to the lack of consensus on what truly constitutes citizen science in the field of nursing, and the debates on terminology, classification, and other characteristics, only those studies that explicitly defined the approach used as “citizen science” were included in the study. As a result, articles with a higher degree of individual participation may have been excluded if they were not explicitly classified as CS, but perhaps as community participation or crowdsourcing. The lack of consensus makes it difficult to delineate the boundaries in this search.

## 5. Conclusions

The results suggest that citizen science (CS) can be a valid approach to addressing key aspects within the field of nursing. Nursing research is still scarce and primarily clustered in five areas, with environmental health being prominent. Therefore, there is a need for more work with a citizen science approach that addresses other fields of research.

All studies aim to assess the feasibility of involving citizens in research, although in most studies, it is limited to data collection (contributory), highlighting the need to develop projects involving greater participation (contributory and co-created), with greater capacity to drive knowledge development and individual empowerment. It is necessary to develop new studies involving a larger sample size to facilitate generalizing the findings derived from citizen science. Technology can assist in engaging the population in these studies and in the development of new participation tools. 

The limitations encountered hinder the generalization of the results found in the review. However, the results suggest that the citizen science (CS) approach can be an appropriate research methodology for projects in the field of health, specifically in nursing. Therefore, more high-quality studies employing these approaches are needed to further expand the benefits of this approach.

## Figures and Tables

**Table 1:** Assessment of the quality of included studies.

	Authors and Year												
Questions	Brickle, 2017 [38]	Tuckett, 2018 [39]	Tran, 2019 [40]	Kim, 2020 [41]	Odunitan, 2020 [42]	Hahn, 2020 [43]	Cardarelli, 2021 [44]	Rowbotham, 2022 [45]	Tuckett, 2022 [46]	Evans-Agnew, 2022 [47]	South, 2022 [48]	Hobensak, 2023 [49]	D’Alonzo, 2023 [50]
Is there congruity between the stated philosophical perspective and the research methodology?	✔	✔	✔	✔	✔	✔	✔	✔	✔	✔	✔	✔	✔
2. Is there congruity between the research methodology and the research question or objectives?	✔	✔	✔	✔	✔	✔	✔	✔	✔	✔	✔	✔	✔
3. Is there congruity between the research methodology and the methods used to collect data?	✔	✔	✔	✔	✔	✔	✔	✔	✔	✔	✖	✔	✔
4. Is there congruity between the research methodology and the representation and analysis of data?	✔	✔	✔	✔	✔	✖	✔	✔	✔	✔	✔	✔	✔
5. Is there congruity between the research methodology and the interpretation of results?	✔	✔	✔	✔	✔	✔	✔	✔	✔	✔	✔	✔	✔
6. Is there a statement locating the researcher culturally or theoretically?	✔	✔	✔	✔	✔	✔	✔	✖	✖	✔	✔	✖	✔
7. Is the influence of the researcher on the research, and vice- versa, addressed?	✔	✔	✔	✔	✔	NS	✖	✖	✖	✖	✔	✔	✖
8. Are participants, and their voices, adequately represented?	✔	✔	✔	✔	✔	✔	✔	✔	✔	✔	✖	✖	✔
9. Is the research ethical according to current criteria or, for recent studies, and is there evidence of ethical approval by an appropriate body?	NS	✔	✔	NS	✔	NS	NS	✔	✔	NS	✔	✔	NS
10. Do the conclusions drawn in the research report flow from the analysis, or interpretation, of the data?	✔	✔	✔	✔	✔	✔	✔	✔	✔	✔	✔	✔	✔

Note: NS means not recognized.

**Table 2:** Study Characteristics.

First Author (Year)	Design	Aim	Participants (n)	Conclusions
Brickle, MB., (2017) [38]	Photovoice methodology	To involve young people in researching their community’s smoke pollution to turn them into advocates for environmental justice.	10 young people 13–17 years old	Citizen science (CS) promotes the empowerment of young people in participatory action research. It increases critical awareness.
Tuckett, A., (2018) [39]	Exploratory study, Our Voice citizen science approach	To engage and empower participants to document their lived experiences and drive positive change in their local environment (physical health and mental well-being).	8 adults 65 years and older	The sample involved has actively advocated for the implementation of solutions for the benefit of their whole community.
Tran, VT., (2019) [40]	Qualitative methodology, completion of questionnaire (open-ended question)	To identify and list improvement ideas in the care of patients with chronic diseases, from the patients’ perspective.	1636 patients with chronic disease Mean age: 49 years (SD = 14.4)	Patients proposed many ideas to improve their healthcare, from the content of consultations to the organization of hospitals. Citizen science as a method to leverage patients’ practical knowledge.
Kim, KK., (2020) [41]	Survey-based qualitative research	Engage youth as leaders in addressing food security, foster interest in research, and familiarize participants with mobile technology as tools for change. To assess community health and food security using a mobile application to inform the prioritization of community services and resources.	12 young people 13–18 years old	Young people can engage as citizen scientists, generate meaningful data, and contribute from their perspective to community food security discussions. Basis for the development of several funded projects.
Odunitan-Wayas, FA. (2020) [42]	Exploratory study, Our Voice citizen science approach	To assess the feasibility of citizen science to identify and address barriers to physical activity (PA) in a low-income South African community.	11 participants 21–45 years old	Opportunity to collaborate in formulating relevant solutions to improve their local environment.
Hahn, EJ., (2020) [43]	Descriptive study (sample collection)	To assess the feasibility of the CS approach to increase awareness of home radon testing in rural areas of Kentucky and suburbs of Ohio. Secondary objective was to assess the agreement between indoor radon values and outdoor measurements using soil samples in Kentucky.	27 students (under 18 years old)	Engaging young people in a CS project to increase radon testing. The measurements obtained by them were valid
Cardarelli, KM., (2021) [44]	Photovoice, Environmental sampling	To compare critical components of youth engagement in environmental health promotion, scientific communication, advocacy, and research using the youth empowerment framework; and (2) to highlight individual, organizational, and community challenges and potential solutions for engaging young people in environmental health research and advocacy efforts.	60	Youth involvement in research and health promotion will not only develop the next generation of scientists but will also advance environmental health sciences.
Rowbotham, S., (2022) [45]	Pilot study (Photography and Description of Facilities)	To explore the feasibility of using a citizen science approach to collect data on workplace support for breastfeeding.	37 >18 years	Feasibility of using a citizen science approach to gather data on key features of the physical space and workplace culture that facilitated or hindered women in combining breastfeeding and work.
Tuckett, A., (2022) [46]	Qualitative research-action study using the Our Voice method	To assess the citizen science approach in a local food security initiative for older adults and understand the strengths and weaknesses of the initiative. Also, to explore the potential of citizen scientists to bring about change.	13 older adults receiving retirement pensions.	There is viability in using citizen science to evaluate and enhance a local food security initiative. Participating older adults were able to achieve improvements in the initiative that will benefit the wider community. Findings lay the groundwork for expanding citizen science.
Evans-Agnew, R.A., (2022) [47]	Qualitative research-action methodology, Photovoice	Describe how an environmental justice project on air quality through citizen science can be useful for collecting, analyzing, displaying, and evaluating indoor threats from volatile organic chemicals.	15 youths aged 10 to 17	Involving youths in collaborative analysis and dissemination can be a useful way to promote social change. Across generations, youths developed greater solidarity with adults in seeking environmental justice. Process evaluation shows that the desire for continuity and enthusiasm bode well for policy improvement in this community.
South, K., (2022) [48]	Phenomenological qualitative study through interviews	To explore the experiences of COVID-19 symptoms, both suspected and confirmed, among women using the CS mobile application CovidWatcher.	28 women users of CovidWatcher aged between 18 and 83 years	Significant impact of the pandemic on the self-perception of women’s physical and mental health symptoms. Symptoms related to the general stress of living in a pandemic generally affected the participants.
Hobensack, M., (2023) [49]	Combined qualitative phenomenological and descriptive qualitative study	To understand the frequency and predictors of COVID-19 symptom reporting in CovidWatcher, a CS mobile application, among older adults.	1028 participants >18 years	The study highlights the potential of citizen science to support participation in symptom reporting during the early phase of the COVID-19 pandemic. New motivating strategies should be designed to increase participation.
D’Alonzo, KT., (2023) [50]	Qualitative research based on discussions	To design a programme to train community researchers to address obesity and dietary health among Mexican immigrant families. In turn, identifying key components of a successful programme.	11 young students of Mexican descent	The study demonstrates that Mexican-descent adolescents who receive training as researchers can have an impact on promoting healthy lifestyles in their families and communities.

**Table 3:** Assessment of the quality of the CS approach using the CSAT tool.

	D1: Science and Research		D2: Leadership and Participation		D3: Delivery and Data		D4: Results, Evaluation, and Open Data										Total
Authors and Year	1	2	3	4	5	6	7	8	9	10	11	12	13	14	15	16	=
Brickle, MB., et al. (2017) [38]	2	2	2	2	2	2	2	1	1	1	2	2	0	0	1	2	24
Tuckett, A., et al. (2018) [39]	2	2	2	2	2	2	2	2	2	1	1	1	0	2	2	1	26
Tran, VT., et al. (2019) [40]	2	2	2	2	2	2	2	1	2	2	0	0	2	2	2	1	26
Kim, KK., et al. (2020) [41]	2	2	2	2	1	1	2	2	2	2	2	0	0	0	2	2	24
Odunitan-Wayas, FA., et al. (2020) [42]	2	2	2	2	2	1	2	2	1	1	0	0	0	0	2	1	20
Hahn, EJ., et al. (2020) [43]	2	2	2	2	2	1	2	1	1	1	1	0	0	2	2	2	23
Cardarelli, KM., et al. (2021) [44]	2	1	0	1	0	0	2	1	1	0	0	0	0	2	1	0	11
Rowbotham, S., et al. (2022) [45]	2	2	2	2	2	2	2	1	2	2	0	0	0	0	1	1	21
Tuckett, A., et al. (2022) [46]	2	2	2	2	2	2	2	2	2	0	1	2	0	0	2	1	24
Evans-Agnew, R.A., et al. (2022) [47]	2	2	2	2	2	1	2	2	2	2	2	1	0	0	2	1	25
South, K., et al. (2022). [48]	2	1	0	1	2	0	2	0	1	0	0	0	0	2	0	0	11
Hobensack, M., et al. (2023). [49]	2	1	2	1	1	2	2	0	0	0	0	0	0	0	0	1	12
D’Alonzo, KT., et al. (2023) [50]	2	2	1	1	1	0	2	1	1	2	2	0	0	0	1	0	16

Legend table: 1. Is there a clear statement of the aims, objectives, or goals of the study? 2. Is it clear that the study used a citizen science approach? 3. Is the degree of active engagement or participation of citizens identified clearly by the study? 4. Are the roles, responsibilities, and type of partnership between citizens, scientists, and stakeholders identified and transparent? 5. Is the extent to which citizen scientists are actively engaged or collaborate in data collection, analysis, and use/dissemination clear? 6. Are citizen science data limitations or biases considered by the study? 7. Are the main findings of the study clearly described? 8. Are the study’s outcomes a direct result from the data-driven strategies and solutions generated by the citizen scientists? 9. Do the outcomes of the study have “real world” decision-making implications or impact? 10. Does the study report intention to track and/or tracking of long-term impacts, changes or “ripple effects” of the study? 11. Does the study report any evaluation of citizen knowledge, attitudes, and actual and/or intended behaviors? 12. Does the publication report any accessible dissemination plans or intentional mechanism for sharing the study and its outcomes with citizens? 13. Are citizens invited to review or participate in the study’s publication process? 14. Are the study’s results and outcomes published in an open access format and/or shared in a publicly accessible format? 15. Are citizen scientists acknowledged in the study’s results and publications? 16. Does the publication provide any critical evaluation of the study, methods, and/or examination of its limitations? Points: yes = 2; unclear = 1; no = 0.

## Public Involvement Statement

Not applicable, no public involvement in this research.

## Guidelines and Standards Statement

This manuscript was drafted according to the PRISMA 2020 Statement.
